# Biomass allocation in response to accession recognition in *Arabidopsis thaliana* depends on nutrient availability and plant age

**DOI:** 10.1080/15592324.2021.2004025

**Published:** 2022-01-20

**Authors:** Thiara S. Bento, Mark B. Moffett, Danilo C. Centeno, Anna Paula D. Scrocco, Austin Fox, Andrew G. Palmer

**Affiliations:** a Department of Ocean Engineering and Marine Sciences, Florida Institute of Technology, Melbourne, Florida, USA; b Department of Aerospace, Physics and Space Sciences, Florida Institute of Technology, Melbourne, Florida, USA; c Centro de Ciências Naturais E Humanas, Universidade Federal Do Abc, São Bernardo Do Campo, Brazil; d Instituto de Botânica de São Paulo, Núcleo de Pesquisa Em Fisiologia E Bioquímica de Plantas, Avenida Miguel Stéfano, Brazil; e Department of Biomedical and Chemical Engineering and Science, Florida Institute of Technology, Melbourne, Florida, USA; f Aldrin Space Institute, Florida Institute of Technology, Melbourne, Florida, USA

**Keywords:** Kin recognition, metabolomics, Arabidopsis thaliana, nutrient variability, identity recognition

## Abstract

Many organisms have evolved to identify and respond to differences in genetic relatedness between conspecifics, allowing them to select between competitive and facilitative strategies to improve fitness. Due to their sessile nature, plants frequently draw from the same pool of nutrients, and the ability to limit competition between closely related conspecifics would be advantageous. Studies with *Arabidopsis thaliana* have confirmed that plants can detect variations at the accession level and alter their root system architecture (RSA) in response, presumably for regulating nutrient uptake. The phenotypic impact of this accession-recognition on the RSA is influenced by nutrient availability, underscoring the importance of plant-plant recognition in their growth and fitness. Thus far, these observations have been limited to short-term studies (<21 days) of only the RSA of this model angiosperm. Here we exploit nutrient-mediated regulation of accession-recognition to observe how this plant-plant recognition phenomenon influences growth from germination to flowering in *A. thaliana*. Our work identifies root and shoot traits that are affected by nutrient-mediated accession recognition. By coupling phenotypic assays to mass spectrometry-based studies of primary metabolite distribution, we provide preliminary insight into the biochemical underpinnings of the changes observed during these plant-plant responses. Most notably that late-stage changes in sucrose metabolism in members of the same accession drove early flowering. This work underscores the need to evaluate accession-recognition under the context of nutrient availability and consider responses throughout the plant’s life, not simply at the earliest stages of interaction.

## Introduction

Due to their sessile nature, plants compete for key resources (e.g., light, water, and soil nutrients) with their neighbors, potentially facing negative impacts on growth if they deplete one or more of these resources too quickly. As a result, nutrient availability is one of the main drivers of competitive and cooperative plant-plant interactions between individuals occupying a similar niche.^1–3^Abiotic cues, like nutrient availability, are integrated with biotic ones, like the identity of neighboring plants, to direct biomass allocation to specific organs to mediate the uptake of limiting resources. Such developmental plasticity is easily observed in changes in either the root system architecture (RSA) or the aerial portion of the plant (stems and leaves), which are responsible for the uptake of nutrients and water, or to provide surface area for increased photosynthesis, respectively.^4^ In plants with limited mobility coupled to limited seed dispersal, assemblages form high in both density and genetic relatedness.^5,6^ When living in such dense related groups, the ability to distinguish the extent of relatedness between members of the same species (conspecifics) likely plays an important role in population structure. Generally speaking, the ability to distinguish between genetically related and unrelated conspecifics is known as kin recognition and typically focuses on immediate relations (parents, siblings, etc.).^7,8^ However, in plants, the extent to which genetic-relatedness between conspecifics is recognized varies by species, from the specific recognition of members of the same seed pod (kin) to those which are merely members of the same accession. For example, the model angiosperm *Arabidopsis thaliana* can distinguish members of different accessions, but all members of the same accession appear to initiate similar responses whether they are siblings or unrelated.^9^ Ultimately, the more appropriate term ‘identity recognition can be used to distinguish between ‘degrees’ of relatedness (kin, accession, etc.) and their impact on growth and development.^10^

The existence of such identity recognition strategies at the level of kin or between members of the same accession is predicted by kin selection theory which proposes that an individual’s cooperative behavior toward relatives can positively impact plant growth and fitness. Such cooperative behaviors ultimately affect the genetic structure and diversity of a population, typically lowering genetic diversity.^7^ Such narrowing in diversity likely optimizes for traits that allow the population to function cooperatively or minimize resource competition.^11,12^ As a result, the dense assemblages of closely related members of the same species described above should work cooperatively, or at the very least less competitively.^13^ Meanwhile, understanding the mechanisms that regulate identity recognition is important for understanding competition in natural ecosystems and developing novel strategies for improving agricultural yields, especially in monocultures where the majority of the crop are typical of limited genetic variability (same varietal or accession).

Previous studies have confirmed that, like other plant-plant interactions, identity recognition is also influenced by nutrient availability.^14–17^ In general, these studies confirmed that among conspecific pairs of plants, closer relatives allocated significantly less biomass to their RSA than more distant ones under nutrient scarcity conditions. The observed reduction in root biomass suggests a similar reduction in nutrient uptake between more closely related individuals. In the case of *Arabidopsis thaliana*, our study confirmed that nutrient-mediated identity recognition was limited to members of the same accession as the response of seeds from the same silique (seed pod) were indistinguishable from those from other plants of the same ecotype.^9^

Our prior studies observed the relationship between nutrient availability and kin recognition only over the first three weeks of seedling growth (21 days post-germination) using solid-phase media. These studies established that ‘low’ nutrients increased lateral root number while increased genetic relatedness reduced this effect.^9^However, plants must integrate nutrient availability over their lifetime, raising the question of whether this impact on identity recognition persists throughout their life cycle. Herein, we have investigated the effect of kin recognition and nutrient availability on the growth of *A. thaliana* at later developmental stages (*e.g*., rosette area, stem height, leaf area, aboveground biomass, root: shoot ratio, root length, root biomass, as well as reproductive timing) and the changes in primary metabolism that underpin these changes. Specifically, we investigated how the *A. thaliana* ecotype (Col-0) responded to sustained growth in the presence of neighbors of either the same or different ecotypes, under ‘regular’ (0.5xMS) or ‘low’ (0.1xMS) nutrient conditions. Given that identity recognition in *A. thaliana* appears restricted to differences in ecotype and not between seedlings from different parental lines, we will use the term ‘kin’ for members of the same ecotype and ‘stranger’ (abbreviated STR herein) for those of different ecotypes. This is consistent with other studies in this area.^18,19^

**Figure 1.**
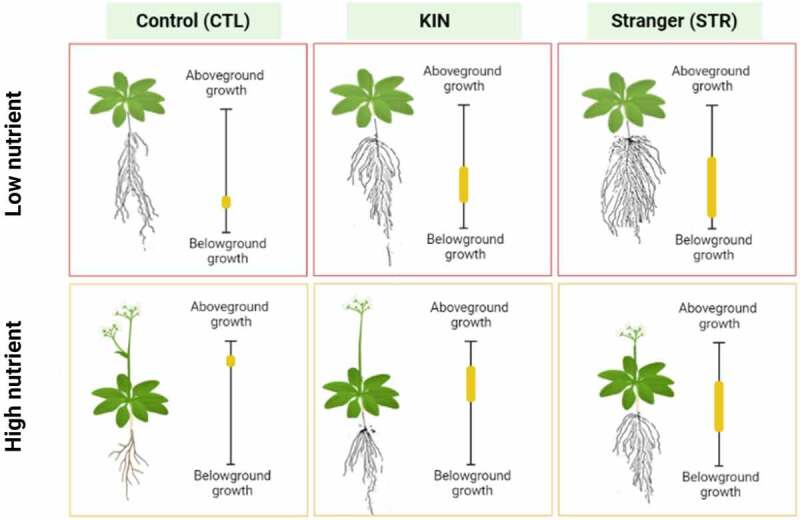
*Proposed identity recognition responses of Col-0 seedlings at later developmental stages*. The position of the yellow bar indicates whether biomass allocation will be to roots or shoots, while the size of the bar represents the intensity of the response. CTL plants under low nutrient conditions will favor root over shoot biomass allocation. In contrast, under regular (0.5x MS) nutrient conditions, plants will allocate more biomass to the shoots. KIN and STR (Stranger) pairings should show a similar trend of increased root biomass relative to shoot biomass under low nutrient conditions. Increased root allocation under low nutrient conditions should follow a trend of CTL< KIN < STR. In other words, as genetic relatedness increases so does root allocation under low nutrient conditions. Figure created with BioRender.com.

We hypothesized that identity recognition in *A. thaliana* plants would allocate more resources to root biomass under lower nutrient conditions, regardless of the growth stage. More specifically, we predicted that kin plants under nutrient stress would show increased root biomass relative to solitary *A. thaliana* controls. Expanding on this hypothesis, we also propose that under nutrient stress, kin plants will show less root biomass allocation in response to neighbor identity than non-relative plants (Stranger) (Figure 1). These results would be consistent with the data we previously acquired on seedling growth under similar conditions.

Changes in biomass allocation in plants in response to varying nutrient availability are driven by metabolic changes in the shoot and an adjustment of carbohydrate transport to the roots.^20^ In this context, we hypothesized that several primary metabolites, mainly sugars, would show increased allocation to the roots during interactions with members of different accessions (stranger) due to increased root distribution as a function of nutrient availability. This biochemical approach is essential to elucidate the strategies used by *A. thaliana* during kin recognition, as changes in metabolite localization and biochemical pathways are key indicators of adaptive responses. Our findings confirm that identity recognition is not a static response in plants but that strategies change due to plant age and nutrient availability. These findings underscore the complexity of the kin recognition process and the potential of plants to manipulate these pathways to adapt over time. Finally, we discuss how identity recognition responses may vary between annuals and perennials to optimize nutrient acquisition in dense, closely related populations.

## Materials and methods

### Design and printing of 3D hydroponic boxes

We custom-designed and 3D-printed a hydroponic system to facilitate the study of plant-plant interactions (e.g., kin vs. stranger) in small plants like *A. thaliana*. Specifically, we sought to create a system that allowed two plants to be connected and share exudates through water channels while eliminating mechanical contact between them. Our system consists of two chambers (25 mL capacity each) connected by a 4 cm channel to facilitate water flow. Single boxes were used as CTL. Plant holders were designed to hold agar plugs to minimize stress during initial growth and transfer to hydroponic boxes. Bubblers were also designed to avoid root anoxia.^21^

**Figure 2.**
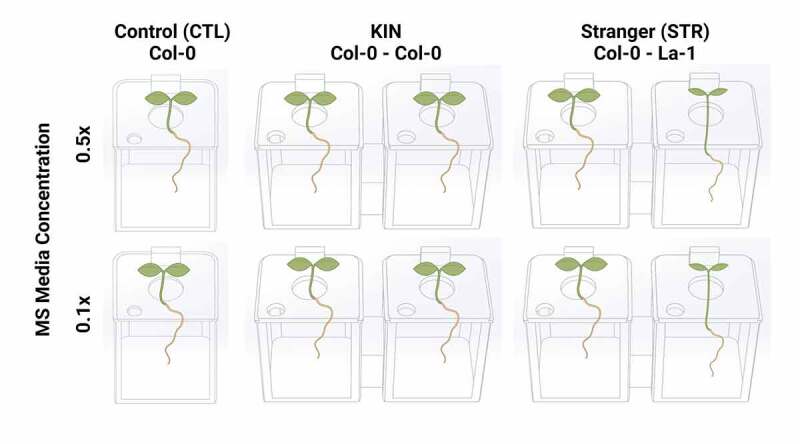
*Experimental design schematic for the identity recognition studies performed in 3D-printed hydroponic boxes. A. thaliana* genotypes Col-0 and La-1 were grown in hydroponic conditions with either 0.1x or 0.5x MS media. Each nutrient condition included CTL (n = 12), KIN (n = 8), and STR (n = 8) . There were a total of 80 boxes and 112 plants. All plants were grown under similar temperatures (23°C, 16:8 hr day:night) created with BioRender.com.

All boxes and accessories were designed using Autodesk® Tinkercad® software and exported as STL (StereoLithography) files (Figure 2). STL files were processed using Ultimaker Cura 4.8.0 printing software and exported as gcode files (See Supplementary material). Hydroponic systems were printed on a WANHAO® Duplicator 4 desktop 3D printer with PLA filament. A total of 48 coupled boxes and single boxes were printed, with a matching number of lids, plant holders, and bubblers. The ability to custom print small boxes and lids allowed us to grow plants in volumes as low as 25 mL. It also allowed us to perform controlled experiments to observe individual plants’ responses under variable nutrient conditions.

### Plant description

*A. thaliana* is an annual, self-fertilizing plant with limited seed dispersal and occupies a wide range of environmental conditions across all continents.^22,23^ It is also a well-accepted model system for plant development, immunology, and physiology. In natural populations, individuals are often surrounded by plants derived from the same parent, and plant interactions are most likely between closely related individuals. As a ruderal species adapted to disturbed conditions, it is also highly sensitive to interspecific competition, showing a reduction in size, flowering time, and seed production.^24^ Altogether, these factors suggest this may be an ideal plant for observing both inter-and intra- specific strategies for competitive and facilitative responses among annual plants and why we continue to employ it as a model system in kin recognition studies.

### Seed sterilization

*A. thaliana* seeds (Columbia-0 ecotype, Col-0, and Landsberg erecta, La-1, purchased from Lehle seeds) were surface-sterilized in a 2% sodium hypochlorite solution for 5 minutes, followed by a 3-minute exposure to 90% ethanol. Between each treatment, seeds were rinsed in triplicate with an equal volume of sterile deionized water. Following surface sterilization, seeds were transferred to the 3D-printed plant holders filled with agar media (0.1 and 0.5x Murashige and Skoog medium (MS), 8 g/L agar, pH: 5.5).

### Plant growth

*A. thaliana* (Col-0 ecotype) was used as the reference plant for all experiments, while the *A. thaliana* (La-1 ecotype) was designated as the non-related neighbor. Three distinct assortments were employed in this study: (i) Col-0 seedlings paired with Col-0 designated as ‘Kin’ (KIN)(coupled boxes)(N=8), (ii) Col-0 seedlings paired with La-1, designated as ‘Stranger’ (STR) (coupled-boxes)(N=8), and (iii) Col-0 seedlings grown in isolation, designated as ‘Controls’ (CTL) (single box) (N=12)(Figure 2). There were 80 boxes and 112 plants in total. Each seedling was grown in our custom-designed boxes with 50 mL for the coupled boxes or 25 mL for the single boxes of the indicated nutrient concentration growth solution (low or regular MS) for eight weeks at ambient temperature (≈23°C) with a 16:8 h day: night cycle. An equivalent volume of new sterile MS liquid media was added to each box every two days (3 ml/day).

### Collection and analysis of plant material

At the end of the eight-week growth period, Col-0 plants were harvested, and shoot, or root biomass were collected (fresh and dry). Collected digital images of the plants were used to classify the developmental growth stages according to 25. The leaf area was determined using ImageJ from the rosette’s digital photographs (26).

### Plant metabolomics (Gas chromatography-mass spectrometry) study

To study changes in primary metabolism, shoot and root portions of each Col-0 plant were separated, frozen in liquid nitrogen, ground to a fine powder, and lyophilized. Primary metabolites were extracted from the lyophilized shoot or root samples in methanol:chloroform: water [12:5:1] solution and spiked with an internal standard (adonitol, 0.2 mg/ml).^27^ The samples were incubated at 60°C for 30 min, centrifuged, and 350 µl of supernatant was collected. Deionized water was added to the collected supernatant, and the resulting organic phase was collected for further analysis. The organic phase was dried under vacuum and *N-O*-bis (trimethylsilyl), trifluoracetamide (BSTFA) derivatized by reaction with pyridine, and methoxyamine hydrochloride (20 mg/ml^−26^ pyridine).^28^ Gas Chromatography-Mass Spectrometry (GC/MS) was performed on an Agilent GC 6890-coupled to an Agilent MSD 5973 N quadrupole mass-spectrometer(Agilent Technologies, USA), modified from 29, where a DB-5 ms column was used in an oven with an initial temperature of 70°C and a final temperature of 280°C with a 5°C/min increasing ramp (30 min total). The total ion chromatogram (TIC) and mass spectra were analyzed using Chem Station (Agilent) software. The detected peaks were identified using the NIST 08 Spectral Library and confirmed by Kovats indices. The plant metabolomics data were analyzed using MetaboAnalyst 5.0 for comprehensive and integrative metabolomics data analysis^30^

### Statistical analysis

Statistical analyses were performed using two-way ANOVA for normally distributed data to calculate the statistically significant differences between the nutrient availability and neighbor identity. When appropriate, post hoc mean comparisons were conducted using Tukey’s test at 5% probability (P ≤ 0.05). Normality was assessed using the Shapiro–Wilk test, assuming normally distributed data for *p*-value > 0.05 using GraphPad Prism (v6.0.0 www.graphpad.com). Metabolomic analysis was performed based on the normalized peak tables using the corresponding functions in MetaboAnalystR 5.0. Principal component analysis (PCA) was applied for outlier detection. Differences in the metabolic profiles of CTL, KIN and STR under nutrient availability were investigated using discriminant analysis (PLS-DA). Because structured noise was not detected in the models, variable influence on projection score (VIP) was used for ranking the predictors within the stability selection procedure. In addition, a heatmap of hierarchical cluster analysis was constructed to present the results of identified metabolites in roots and shoots of *A. thaliana*. It can be used to discover clustering patterns in the datasets. Heatmap was constructed with MetaboAnalyst 5.0. (https://www.metaboanalyst.ca) using peak areas data (*.csv format) from all compounds detected.

## Results

### Plant biomass allocation during kin recognition changed as a function of age, NOT nutrient availability

**Figure 3.**
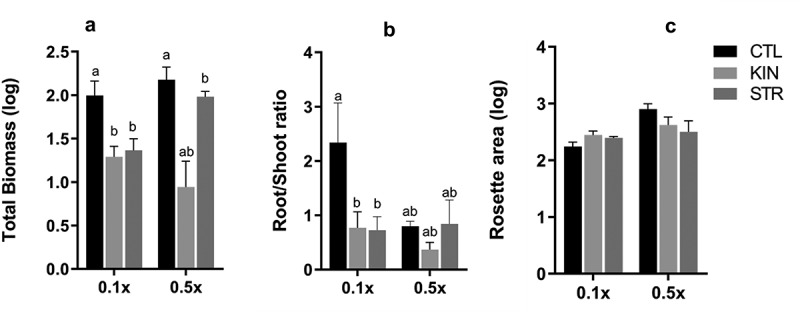
*Changes in morphological growth of* A. thaliana *(Col-0) plants in response to a neighbor under nutrient availability in the hydroponic system*. Identity recognition study was run for 8 weeks at which time samples were harvested and evaluated for phenotypic changes and metabolite isolation of Col-0 seedlings. (a) Total biomass of plants in each treatment. (b) The root-to-shoot ratio of the plants from each treatment (c) Rosette area of the plants growing under 0.1 and 0.5x of nutrient availability. Letters indicate statistically significant differences in samples based on Tukey’s posthoc test (*p*-value < 0.05).

To determine if the synergistic influences of nutrient availability and neighbor identity on plant growth and development persisted throughout a plant’s life, we investigated how the *A. thaliana* ecotype (Col-0) responded to sustained growth in the presence of neighbors of either the same (KIN) or different (‘stranger’ or STR) ecotypes, under ‘regular’ (0.5xMS) or ‘low’ (0.1xMS) nutrient conditions. To accommodate the protracted study required here, we developed a 3D-printed hydroponic system capable of accommodating *A. thaliana* from seed to harvest (See Methods). Samples were grown for eight weeks, then evaluated for the following competitive traits: above/belowground biomass, root-to-shoot ratio, and rosette area (Figure 3).

As seen in Figure 3a, neighbor identity significantly impacted total plant biomass in low and regular nutrient conditions ((P < .0001, 2-way ANOVA). Under low nutrient conditions, the total biomass of CTL plants was significantly greater than both KIN and STR plants (*P* < .05), with 30–35% greater biomass than plants in the presence of a neighbor. Conversely, no significant difference in biomass accumulation was observed under low-nutrient conditions between KIN and STR (*P* = .9514). Under regular nutrient conditions, CTL plant biomass was significantly (38%) higher than KIN plants (*P* = .0001) but not STR plants (*P* = .8011). Indeed, the total biomass of KIN plants was 31% less than STR plants (*P* = .0063) (Figure 3a).

Similarly, both nutrient availability and neighbor identity affected the root-shoot ratio of plants in this study (*P* = .0488, See Figure 3). The root-shoot ratio of CTL under low nutrient conditions was ≈70% greater than KIN (*P* = .0067) and STR (*P* = .0037) plants, respectively. No difference in the root-shoot ratio between CTL, KIN, and STR was observed for regular nutrient conditions (Figure 3b). This data suggests that the presence of a neighbor combined with nutrient availability caused a shift to aboveground growth in older plants rather than roots, as seen in seedlings.

Conversely, no role for identity recognition in the rosetta area was observed. Rosette area was, however, significantly affected by nutrient availability, with an average reduction of 15% under low nutrient conditions compared to regular nutrients conditions (*P* = .0431, Figure 3c).

### Kin recognition can alter developmental timing under nutrient limiting conditions in *A. thaliana*

**Figure 4.**
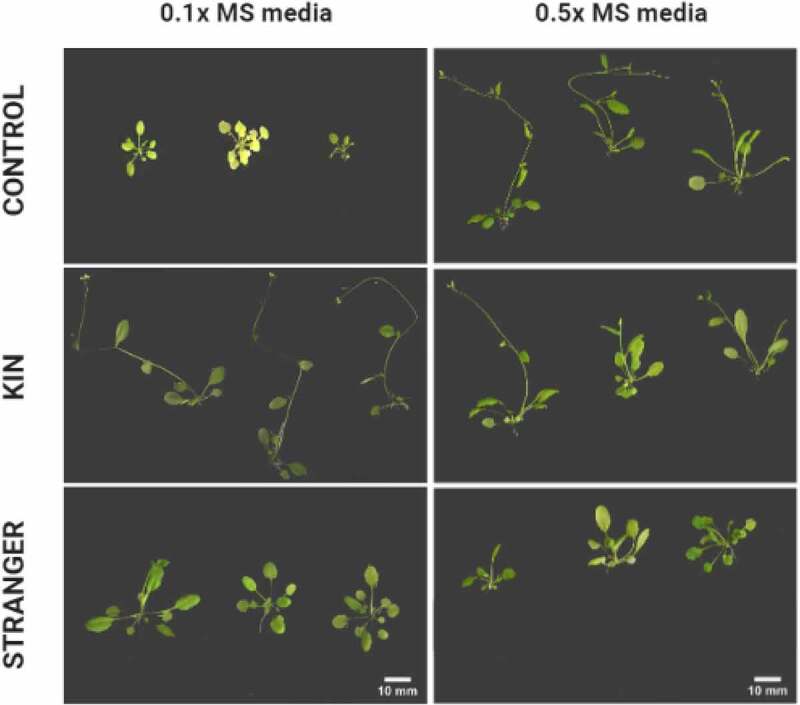
*The effects of neighbor identity on the developmental growth stage of* A. thaliana. Images of Col-0 seedlings grown under low (0.1xMS) or regular (0.5xMS) concentrations of nutrients and in the presence or absence of a similar (KIN) or more distant (STR) relative of *A. thaliana*. Created with BioRender.com.

An unexpected and previously unidentified change in the developmental timing of Col-0 plants in response to neighbor identity was observed (Figure 4, Table S3). Using the classification stages developed by^31^ we observed early flowering development in KIN plants under low nutrient conditions. Specifically, Col-0 plants growing with kin developed their first flower (principal stage 6) during the time of this study as compared to CTL and STR treatments in which no flowers were observed. CTL plants growing under normal nutrient conditions could mimic the KIN phenotype under low nutrient conditions and achieve flowering.

### Metabolic changes in response to kin recognition and nutrient deficiency in *A. thaliana*

**Figure 5.**
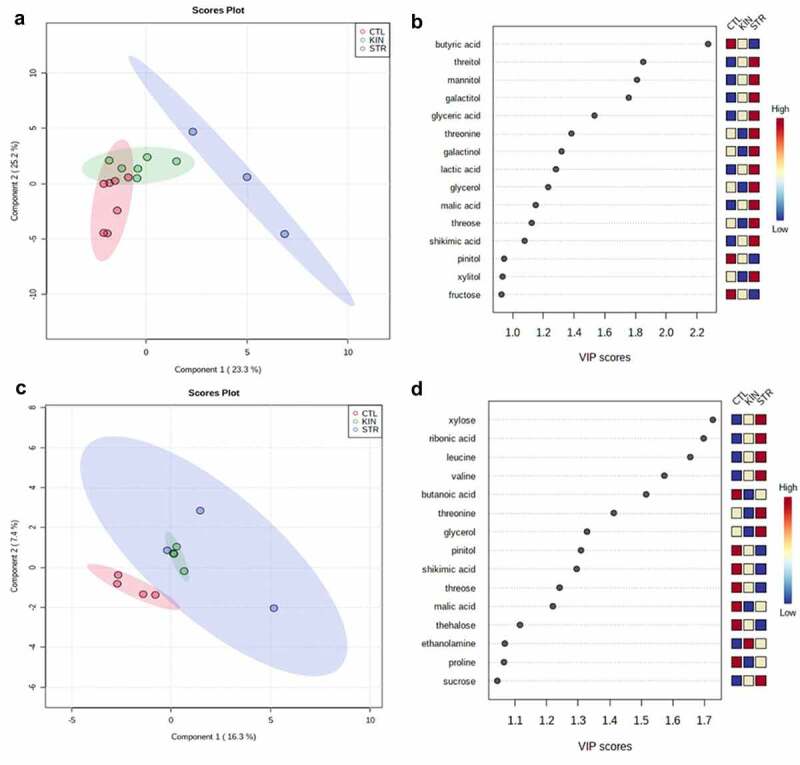
*Primary metabolism changes based on neighbor identity under low (0.1xMS) nutrient conditions*. Partial least discriminant analysis (PLS-DA) was performed to identify important features (metabolites) in the shoots and roots growing under 0.1xMS nutrient deficiency. (a) 0.1x nutrient solution scores plots of CTL, KIN and STR shoots. (b) 0.1x nutrient solution VIP score values of CTL, KIN and STR shoots. (c) 0.1x nutrient solution scores plots of CTL, KIN and STR roots. (d) 0.1x nutrient solution VIP score values of CTL, KIN and STR roots. Colored boxes (right) indicate the relative concentrations of the corresponding metabolite in each group.

**Figure 6.**
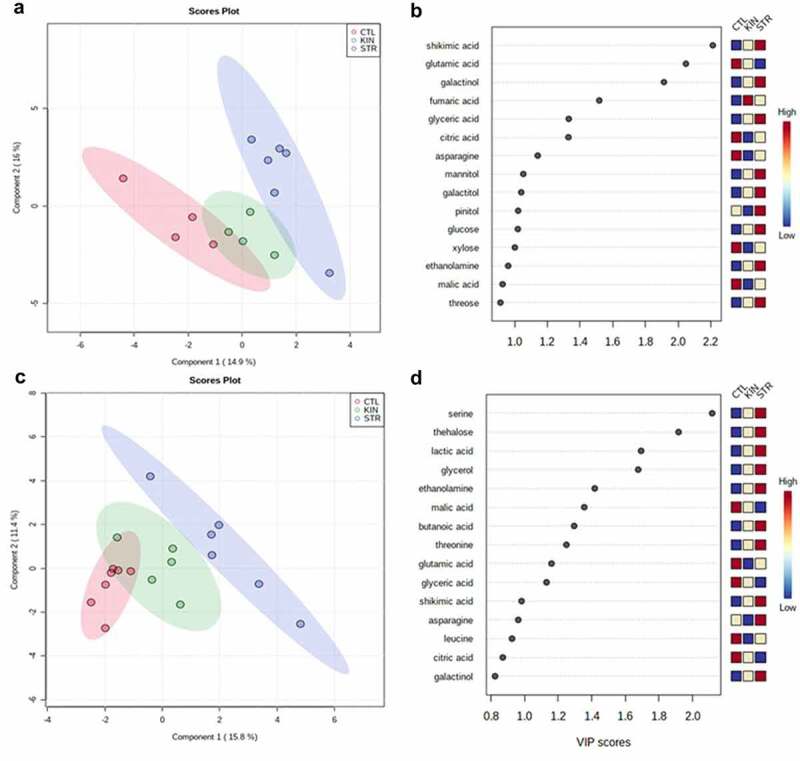
*Primary metabolism changes based on neighbor identity under normal (0.5xMS) nutrient conditions*. Partial least discriminant analysis (PLS-DA) was performed to identify important features (metabolites) in the shoots and roots growing under 0.1xMS nutrient deficiency. (a) 0.5x nutrient solution scores plots of CTL, KIN and STR shoots. (b) 0.5xMS nutrient solution VIP score values of CTL, KIN and STR shoots. (c) 0.5x nutrient solution scores plots of CTL, KIN and STR roots. (d) 0.5x nutrient solution VIP score values of CTL, KIN and STR roots. The colored boxes (right) indicate the relative concentrations of the corresponding metabolite in each group.

Understanding the changes in metabolic profiles that ultimately drive the altered biomass observed during identity recognition can provide insight into the biochemical underpinnings of this phenomenon. We, therefore, profiled the shoot and root primary metabolism of the plants using GC-MS to identify the metabolic changes related to growth (Table S1). A partial least discriminant analysis (PLS-DA), as well as a heatmap of the 36 identified metabolites, were analyzed in the shoots and roots under kin interactions at low and regular nutrient availability. This approach provides a global view of the metabolic changes occurring during kin recognition interactions under various nutrient conditions. As shown in Figures 5 and 6, shoots and roots of CTL, KIN and STR under both nutrient conditions were separated in the PLS-DA score plot with two principal components (PCs), despite the minor overlap. These results indicate that there is a metabolic change between the treatments when compared to control. Of those 36 primary metabolites identified, organic acids were the most common, followed by sugar alcohols, amino acids, and sugars (Figures 5 and 6).

### Identification of the kin recognition responsive metabolites in *A. thaliana* shoots and roots under nutrient availability

As shown in Figure 5a, low nutrient conditions and the presence of an STR plant had a significant effect on the metabolic profile of the shoots of the plants when compared to kin and CTL. The variable importance in the projection (VIP) from the PLS-DA was used to identify the most relevant metabolites for each treatment.^32^ For this analysis, values greater than 1 were considered the most relevant metabolites for explaining observed responses.

Utilizing the established VIP values, 12 metabolites – including four organic acids, two amino acids, four sugar alcohols, and two sugars, were identified as significantly discriminant for shoots growing under low nutrient conditions (Figure 5b). The most common metabolites to be altered in STR-treated shoots were sugars, sugar alcohols and organic acids. Compared to the CTL plants, seven metabolites: shikimic acid, glyceric acid, galactinol, mannitol, galactitol, pinitol, and glucose all increased in concentration, while four metabolites: citric acid, glutamic acid, asparagine, and xylose, decreased in the shoots of STR grown plants. KIN-grown plants generally showed a reduction in these metabolites relative to STR treatments, except for fumaric acid.

Ten metabolites, mainly organic acids and amino acids, were identified as the most relevant metabolites based on the VIP values for roots growing on low nutrient solutions. In the roots, the accumulation of serine, ethanolamine, threonine, lactic acid, trehalose, and glycerol was higher in STR treatments than CTL and kin (Figure 5d). Consistent with PLS-DA score plots results, different but overlapping metabolite-response to kin interactions was observed under regular nutrient conditions (Figure 6a). There were no differences in the metabolic profile of roots between CTL and KIN treatments. Both treatments showed an accumulation of gluconic acid, galactonic acid, glutamic acid, asparagine, and fructose (Figure 6b).

Compared to CTL and KIN, 11 metabolites increased (threitol, mannitol, galactinol, glycerol, threose, shikimic acid, glyceric acid, lactic acid, malic acid and threonine) in levels in the shoots of STR plants (Figure 6b). The accumulation of sugar alcohols was also the main difference observed in the shoots of the STR plants. Meanwhile, on the roots, there was no difference in the metabolic profile between KIN and STR treatments (Figure 6c). Plants increased the levels of xylose, sucrose, ribonic acid, leucine, valine and ethanolamine, and decreased the levels of butanoic acid, pinitol, shikimic acid, threose, malic acid, trehalose and proline compared to CTL plants (Figure 6d).

**Figure 7.**
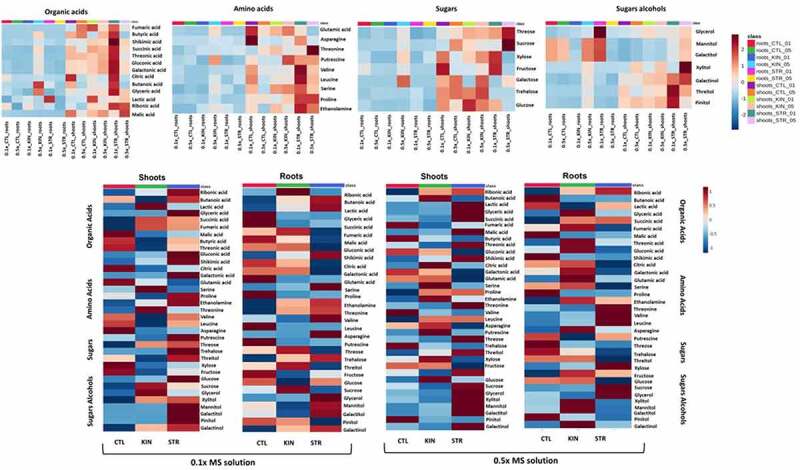
*Heat maps of differential metabolite accumulation between shoots and roots as a result of identity recognition under low and high nutrients*. The average of the normalized data is indicated in shades of red and blue for the increase and decrease, respectively, in the metabolite content. The clustering result is shown as a heatmap (distance measure using Pearson and clustering algorithm using t-test/ANOVA).

To compare the individual metabolites’ changes during kin recognition responses, the heat map of metabolomics data, generated based on the average of the normalized data, was used as a visualization tool (Figure 7). Consistent with PLS-DA results, changes in the metabolite concentrations of plants growing with STR, such as sugar alcohols, were also observed in the heat map. Moreover, metabolites that differentially accumulated in response to altered nutrient conditions were identified. For example, CTL plants showed an accumulation of fructose and glucose in the roots compared to leaves under nutrient deficiency, causing an increase in root: shoot ratio. On the other hand, under nutrient deficiency, the STR plants’ shoots showed a shift in the accumulation of organic acids, amino acids, sugars, and sugars alcohols in general. In summary, we found that the metabolic profiles of KIN and STR plants are substantially different from those observed in CTL plants in both nutrient conditions. Our results suggest that nutrient availability and neighbor identity generate other metabolic effects that contribute to the kin recognition responses observed in Arabidopsis.

## Discussion

Identity recognition in plants allows these sessile organisms to regulate competitive and cooperative behaviors based on the genetic relatedness of their neighbors. Responses to identity recognition exploit plants’ developmental plasticity, allowing them to alter traits such as root system architecture, leaf area, etc., modifying their interactions with their neighbors. Understanding these recognition systems at the biochemical, phenotypic, and genetic levels is crucial to understanding community and population structures within ecosystems and contributing to next-generation farming strategies that seek to optimize nutrient uptake and allocation. Recent studies have confirmed that identity recognition responses are regulated, at least in part, by the availability of the resources they are attempting to optimize these plants to manage.

Research with *Arabidopsis thaliana* has the potential to reveal considerable information about recognition responses, especially in response to nutrient variability. However, to date, all information regarding nutrient availability altering identity recognition in this model angiosperm has been restricted to the seedling stages of growth (<21 days). All the observed effects of identity recognition appear limited to changes in the RSA at these early stages. In the present study, we have evaluated the impact of identity recognition on growth in *A. thaliana* under variable nutrient conditions for an extended period to determine if this phenomenon plays an important role beyond. Specifically, we characterized changes in phenotype and biomass allocation, as well as changes in primary metabolism. Changes in primary metabolism were included in this study to provide a preliminary model of the biochemical changes that are driving the observed phenotypic differences. Consistent with our broad hypothesis that nutrient effects would be conserved throughout the lifespan of *A. thaliana*, we observed that both kin (KIN) and stranger (STR) plants indeed altered biomass allocation relative to CTL plants in this study over their lifetime. However, we observed some unexpected changes in developmental plasticity as a function of nutrient availability. Below we consider our findings in the broader context of nutrient availability and the life history of this model angiosperm.

### Identity recognition responses transition from roots to shoots as *A. thaliana* ages

Under nutrient deficiency, plants frequently increase root growth, presumably to enhance nutrient uptake. As a result, root biomass increases at the expense of shoot growth, as observed by an increase in the root: shoot ratio.^33,19^ *A. thaliana* seedlings have previously been reported to discriminate between members of the same ecotype (defined as ‘kin’ in this and other studies) and different ecotypes (‘stranger’ or ‘STR’) by allocating more biomass to lateral root growth in early developmental stages.^9,34–36^As seen in Figure 3b and Figure3c, we observed that low nutrient conditions only increased the root biomass and root: shoot ratio in CTL plants (Figure 3b and c). Surprisingly, both the KIN and STR treatments allocated more biomass to their shoots, observed by increased leaf and rosette area, as well as a reduced root: shoot ratio Figure 3(a-c) relative to control plants. This is in direct contrast to our results from younger seedlings, in which low nutrient conditions increased RSA (lateral root #) in KIN plants relative to CTL and STRANGER plants relative to KIN with little impact on the shoot portions of the plant.

Our findings are consistent with previous studies of *A. thaliana* plants grown beyond the seedling stage under inter- or intra- specific competition, but not nutrient limiting conditions, which also showed increased rosette growth and leaf area relative to isolated control plants.^24,37,38^ Transcriptomic studies of *A. thaliana* grown with KIN beyond the seedling stage also showed a significant upregulation of genes associated with photosynthesis but not those associated with nutrient uptake.^39^ Taken together, these results suggest that the impact of nutrient availability on identity recognition is more limited as a function of age. Indeed, the reduced root elongation in KIN and STRANGER plants relative to isolated controls suggests that identity recognition may even suppress root allocation under more mature conditions. As Arabidopsis is a relatively small plant, nutrient scarcity should theoretically be rare, and preventing light competition may be important, explaining the relatively increased commitment to shoot resource allocation.^24^

### Kin recognition altered the developmental stages (vegetative vs. reproductive growth) of *A. thaliana*

In annual plants, resource availability and biotic interactions strongly influence the transition from vegetative growth to reproductive maturity.^24^ In our present study, we did not observe a difference in aboveground traits like rosette area between KIN and STRANGER under nutrient scarcity, initially suggesting that the specificity of identity recognition in *A. thaliana* could no longer distinguish between accessions merely that another conspecific plant was present. However, we observed that kin plants under low nutrient conditions developed elongated stems and flowered with greater frequency, unlike their stranger counterparts or isolated controls. Competitive traits, for example, early stem elongation, are often selected for competitors’ presence to facilitate the interception of photons for photosynthesis, indicating that the timing of vegetative growth is essential to the overall success.^24,40^ Flowering time is also a critical life-history trait in annual plants like *Arabidopsis* because it directly influences biomass allocation trade-offs and fitness.^41,42^ In this case, those capable of reproduction observed in kin plants may avoid later competition for nutrient availability during the crucial reproductive phase. In summary, our data showed that kin recognition responses shifted from roots to shoots as *A. thaliana* ages, altering the developmental stage of plants growing with KIN and STR from vegetative to reproductive growth.

### Kin recognition responses caused a shift to metabolites accumulation of the shoots to support reproductive growth in KIN, and STR

How primary metabolism responds to neighbor identity/competition under nutrient scarcity can provide insight into how plants adapt to these external factors beyond merely the observed phenotypic changes.^43,44^ Here we investigated the changes in primary metabolism in response to both kin recognition and nutrient availability. As shown in Figure 5, neighbor identity and nutrient availability affected plant metabolite concentration and allocation relative to isolated plants (Figure 5). Changes in nutrient availability in isolated plants only impacted the allocation of primary metabolites to the roots.

Plants rely on the energy gained via photosynthesis in source organs, mainly mature leaves, to develop new organs such as roots, stems and flowers, referred to as sink organs.^45^ As observed in control plants under low and high nutrient availability, the translocation of photoassimilates depends on source supply and sink demand, i.e., the demand for these primary metabolites was greater in the roots, which supports the increased RSA observed in these controls. Conversely, identity recognition shifted the accumulation of metabolites to source tissues, such as leaves, resulting in an increased photosynthetic area in plants with potential competitive neighbors (i.e., KIN and STR).^46^This accumulation could also support the early shift in reproductive growth (flowering) observed in KIN plants.

Plant growth is limited by photosynthetic and metabolic capacity, nutrient availability, and specific molecular signatures can arise that are diagnostic of particular types of growth (i.e., vegetative vs. reproductive).^47^ For example, developmental stage-specific metabolite signatures in *Arabidopsis thaliana* under nitrogen limitation identified the accumulation of asparagine, an efficient carrier and storage compound for nitrogen, as a marker for vegetative growth stages.^48^ Similarly, under low nutrient conditions, we observed that the shoots of the CTL plant accumulated more asparagine under low nutrient conditions than KIN or STR plants under similar conditions. Since asparagine accumulation was only observed in CTL plants, we propose that isolated plants remained in the vegetative stages while identity recognition, regardless of nutrient availability, pushed plants toward later/reproductive strategies. This is further supported by the increased accumulation of fumaric acid in both KIN and STR treatments. A secondary storage compound for fixed carbon in plants, analogous to starch and sucrose; fumaric acid accumulation in shoots is likely to contribute to the energy necessary for transition to the reproductive phase and changes as a function of plant age.^48,49^

KIN and STR plants also showed an increased accumulation of sugars relative to isolated plants. Specifically, KIN plants showed an accumulation of sucrose in their shoots, while STR plants showed elevated glucose, fructose, and trehalose accumulation in the shoots. The accumulation of these sugars in shoot tissue is indicative of these plants allocating resources toward biomass allocation toward leaves to support growth.^45^ Other studies have also suggested that sucrose accumulation helps to regulate the switch from vegetative to reproductive growth as well as the differentiation of shoot apical meristem (SAM) tissue into flowers,^50,51^ We propose that in STR plants, the observed accumulation of glucose, fructose and trehalose indicates early activation of the trehalose 6-phosphate pathway (T6P) which regulates sucrose accumulation and the transition to vegetative growth and flowering.^50^ Meanwhile, KIN plants that have already moved into the reproductive stage have already accumulated more sucrose to drive flower development explaining the reduced concentrations of these these other sugars in this group.

**Figure 8.**
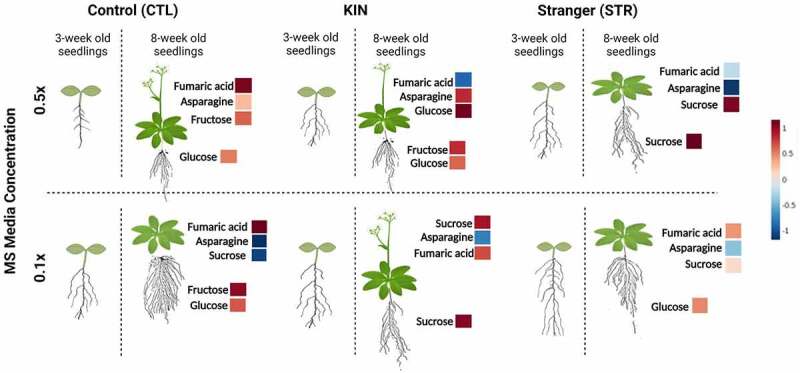
*Biomass allocation in response to kin recognition in A. thaliana is nutrient availability and plant developmental dependent*. We observed that changes in biomass allocation at the later stage in *A. thaliana* were likely driven by changes in the sucrose metabolism favoring early flowering compared to 3-week seedlings. At the early developmental stage, plants allocated biomass in root growth (specifically lateral roots) as a function of nutrient availability.^9^ The bar is indicated in shades of red and blue for the increase and decrease in the metabolite content from the heatmap. Created with BioRender.com.

In conclusion, our results confirm that the metabolic signatures in low and regular nutrient conditions were modified by kin recognition and promoted facilitative effects such as early flowering. Kin recognition responses in plants changed due to age, nutrient availability, and neighbor identity, affecting kin recognition responses in older plants. The primary metabolic profile data showed us that the transition between the adult vegetative and reproductive phases in plants occur much earlier than the visible elongation of the main stem. This metabolic signature of plant development is further influenced by the availability of nutrients, and the distribution shifts in biomass allocation were mainly influenced by kin selection and competitive strategies (Figure 8).

However, it is crucial to consider the natural habitat and lifestyle of the studied plant, e.g., annuals vs. perennials, to put kin recognition strategies in context properly. In our case, *Arabidopsis thaliana* is a fast-growth annual plant growing on disturbed and often nutrient-poor soils. Although early flowering could be a facilitative strategy to avoid later competition for nutrient availability between kin plants in this context, it is also important to emphasize that early flowering in Arabidopsis might result in fewer seeds produced than plants flowering later (when they are larger). Thus, early flowering might not only be beneficial. Perennial plants are expected to invest more into structures that increase their chances of survival over winter. At the same time, annuals must complete their life cycle and reproduce within one growth period and thus allocate their resources accordingly, resulting in more biomass allocation to stems due to a switch to reproduction.^4,52^ The different trade-offs between survival and fecundity for annual and perennial plants could explain the inconsistent results of previous studies about the effects of kin recognition on biomass allocation patterns due to differences in life span characteristics of the species studied.

## Supplementary Material

Supplemental Material
